# Diurnal Choroidal Thickness Changes in Normal Eyes of Turkish People Measured by Spectral Domain Optical Coherence Tomography

**DOI:** 10.1155/2013/687165

**Published:** 2013-03-27

**Authors:** Ozen Ayrancı Osmanbasoglu, Zeynep Alkin, Abdullah Ozkaya, Yavuz Ozpınar, Ahmet Taylan Yazici, Ahmet Demirok

**Affiliations:** ^1^Beyoglu Eye Training and Research Hospital, Retina Clinic, Istanbul 34421, Turkey; ^2^Istanbul Medeniyet Unıversity, Istanbul, Turkey

## Abstract

*Purpose*. To analyse the diurnal variation of central choroidal thickness (CCT) in healthy emetropic patients during working hours. *Methods*. Fifty healthy young emmetrpic volunteers were included in this study. CCT was measured at 9 AM and 4 PM with spectral domain optical coherence tomography (Spectralis, Heidelberg Engineering) with enhanced depth imaging. Diurnal variation of CCT, the correlation between rigth and left eyes and the demographic factors affecting this variation were assessed. *Findings*. The mean CCT at 9 AM and 4 PM was 308.7 ± 64.5 **μ**m and 308.7 ± 62 **μ**m, respectively, with a mean diurnal amplitude of −0.03 ± 14.7 **μ**m, ranging between −55 **μ**m and 47 **μ**m, the difference was statistically insignificant (*P*: 0.9). There were positive correlations between right and left eyes among CCT measurements at 9 AM, 4 PM and the mean amplitude of diurnal change (*r*: 0.65, *P* < 0.01; *r*: 0.60, *P* < 0.01; *r*: 0.45, *P*: 0.00, resp.). There was a statistically significant negative correlation between the magnitude of diurnal change and age (*r*: −0.27, *P*: 0.01). *Conclusion*. Although the mean CCT in the all group does not show significant variation during working hours, the pattern of diurnal variation may vary from person to person according to age, and there is a great harmony between the two eyes.

## 1. Introduction

The choroid is the middle vascular layer of the eye, lying between the retina and sclera. It accounts for 85 percent of ocular blood flow and plays a major role in the oxygenation, nourishment, and viability of retinal pigment epithelium and the outer retinal layers, which have the highest metabolic activity. Choroidal changes such as hyperpermeability, vascular insufficiency, thickening, and thinning were shown to play a role in the pathogenesis of chorioretinal disease. With the recent improvements in technology, Spaide et al. [1] developed the enhanced depth imaging (EDI) technique by using spectral-domain optical coherence tomography (OCT), which enables the visualization and measurement of the choroidal layer. Many studies have been published reporting the choroidal thickness (CT) of patients with various retinal disorders such as central serous chorioretinopathy [2–4], age-related macular degeneration [4–8], polypoidal choroidal vasculopathy [7, 8], Vogt-Koyanagi-Harada syndrome [9], and macular hole [10], multiple evanescent white dot syndrome [11] and diabetic retinopathy [12]. Also, the normative data of choroidal thickness of healthy individuals were reported as a guide for further investigations [13–17]. Therefore, OCT is a valuable tool for the diagnosis and management of chorioretinal diseases as well as helping clinicians understand the underlying pathology.

During the followup and management of patients, repeated OCT evaluations have to be done during different working hours on different visits. Due to its high blood flow and poor autoregulation, choroidal thickness variation could be seen intraindividually. The awareness of circadian changes can lead the management or understanding the mechanism of the diseases. The presence of diurnal fluctuations of human choroidal thickness also has been studied by Usui et al. [18], where the choroidal thickness was measured every two hours during a twenty-four-hour period, but the population was at myopic range. Also Tan and colleagues [19] described the pattern and magnitude of diurnal variation of choroidal thickness of twelve patients whose refractive error ranged between −4.1 and +2.0 s. The purpose of this study is to analyse the diurnal variation of central choroidal thickness in healthy emetropic patients during working hours to determine the affecting factors on diurnal variation in the Turkish population.

## 2. Materials and Method

### 2.1. Subjects

This is a prospective, cross-sectional, observational study performed at Beyoğlu Eyoğlu Eye Training and Research Hospital. The study protocol was approved by the local ethics committee, and all of the patients signed an informed consent. The methodology of the study was designed in accordance with the tenets of the Helsinki Declaration.

Fifty healthy volunteers were included in the study. The exclusion criteria included any ocular illnesses such as the presence of macular abnormality, glaucoma, previous ocular surgery or trauma, amblyopia, visual acuity larger than 0,00 logarithm of the Minimum Angle of Resolution (LogMAR) Unit, refractive error (RE) ranging outside −1.00 +1.00 diopters, axial length (AL) outside 22–25 mm, any systemic vascular diseases such as hypertension, diabetes mellitus and inability to cooperate during screening by OCT examination.

### 2.2. Examination Procedures

All participants underwent a complete ocular examination. All of the procedures were performed once on the same day of OCT examination before the first measurement. Objective refraction was measured by autorefractometer (Canon-RK-F1, serial number: 112213), and best corrected visual acuity was recorded using Snellen charts and converted to LogMAR for analyses. Biomicroscopic and fundoscopic examination with a 90 D lens was performed, and intraocular pressure was measured with a Goldmann applanation tonometer. Ocular axial length was measured using interferometry (IOL-Master, version 918471, model 1322-734; Carl Zeiss Meditec, La Jolla, CA, USA).

OCT scans were performed by the two investigators (either O. A. Osmanbasoglu or Z. Alkin) at 9 AM and 4 PM on an outpatient basis for both eyes. Pupils were dilated with tropicamide 1 percent (Alcon, RØdovre, Denmark) before each OCT examination. Central macular thickness was measured automatically with the software of the system using fast macular examination mode. The choroid was visualized by enhanced depth imaging technique with spectral domain optical coherence tomography (Spectralis OCT, Heidelberg Engineering, Heidelberg, Germany) using a standardised scanning protocol. A single line of 6 mm length centered horizontally on the fovea was used for the visualization of the choroid. To maximise signal-to-noise ratio and reduce speckle noise, the images were acquired in high-resolution protocol. The TruTrack active eye tracking system, which enables the capture of multiple images in the same location, and the automatic real-time (ART) mean function, which combines these images, were used during each image acquisition. ART was set up for 100 frames.The initial (baseline) measurement at 9 AM was set as a reference, and the second scan was done at the same point on the fovea with the eye tracking system.

Choroidal thickness was measured subfoveally using the manual calipers provided by the device software. To avoid interobserver variation, both of the graders measured the choroidal thickness at the same time, and two measurements were taken for each patient in order to assess intrasession variability. Measurement was performed perpendicularly from the outer part of the hyperreflective line (retinal pigment epithelial layer) to the line corresponding to the corneoscleral junction. If the corneoscleral junction could not be identified exactly or the quality of the images was low (below Q 25), the eye was dismissed from the study.

### 2.3. Outcome Measures

This study evaluated the mean choroidal and central retinal thickness at 9 AM and 4 PM and the diurnal change in choroidal thickness. Demographic factors affecting the diurnal variation were also investigated.

### 2.4. Statistical Analysis

Statistical analyses were made using commercially available software SPSS version 16.0 (SPSS Inc., Chicago, IL). For the statistical analyses, the mean (SD) of the differences was calculated. The images were measured twice at the same time by the two observers; the difference of the two measurements was analysed with paired sample *t* test, and intraclass correlation was performed by a Pearson correlation test. Bland-Altman plot was generated to assess agreement of measurements between the two measurements of the same session. Differences in thickness between measurements were plotted against mean choroidal thickness measurements on the graph [20]. A paired sample *t*-test was used to compare the diurnal change of the central subfoveal choroidal and central macular thickness. The correlation coefficient (Pearson correlation) was calculated for the relationship between the subfoveal choroidal and retinal thicknesses. The magnitude of CT change was calculated by extracting CT value that was measured at 4 PM from the value measured at 9 AM. The relationship between age, axial length and the magnitude of diurnal change was analysed with Pearson correlation. To evaluate the effect of sex on diurnal variation, patients were grouped and the differences between the groups were analysed with a *t* test. *P* values of 0.05 were considered statistically significant.

## 3. Results

One hundred eyes of fifty patients (27 female, 23 male) were evaluated. None of the participants had a history of systemic or ocular disease. The measurements of eighty-five eyes (43 right eye, 42 left eye) were eligible for analysis, and fifteen eyes were dismissed from the study because of low image quality and/or inadequate evaluation of corneoscleral junction. The mean age was 45.2 ± 7.4 years. All eyes had the best corrected visual acuity of 0,00 LogMAR Unit, and mean refractive error was +0.50 ±0.25 diopters. Mean axial length was 23.1 ± 0.8 mm. Mean intraocular pressure was 15.3 ± 2 mmHg.  Table 1 shows demographic data.

The mean baseline choroidal thicknesses among eighty-five eyes at 9 AM and 4 PM were 308.7 ± 64.5 *μ*m, and 308.7 ± 62 *μ*m respectively, with a mean diurnal amplitude of −0.03 ± 14.7 *μ*m; the difference was statistically insignificant (*P*: 0.9). Of those patients who had two eyes included in the study (*n*: 35), mean choroidal thickness at 9 AM and 4 PM and the mean diurnal amplitude of the right eyes were 304.9 ± 64 *μ*m, 305.9 ± 59.6 *μ*m, and −0.97 ± 16.6 *μ*m, and the left eyes were 312.5 ± 75.6 *μ*m, 311.6 ± 65 *μ*m and 0.92 ± 12.7 *μ*m, respectively. There were positive correlations between the right and left eyes in the morning and evening choroidal thickness measurements and the mean amplitude of diurnal change (*r*: 0.65, *P* < 0.01; *r*: 0.60, *P* < 0, 01; *r*: 0.45, *P*: 0.00 resp.). Over the same time period, the baseline central macular thickness measurement at 9 AM was 272.2 ± 17 *μ*m, and it did not show significant variation on a paired sample *t* test (*P*: 0.6). The morning and evening central macular and choroidal thickness did not show any significant correlation (*r*: −0.06, *P*: 0.6; *r*: −0.03, *P*: 0.7).  Table 2 summarizes baseline and follow-up measurements.

**Figure 1:**
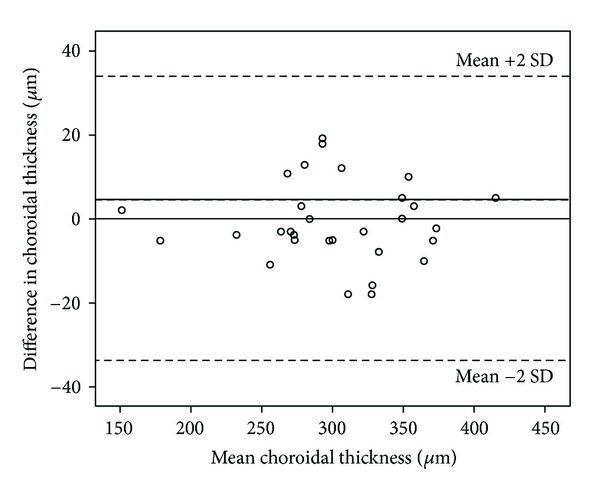
Bland-Altman plot for choroidal thickness. Dotted lines delinate mean and %95 confidence limits of agreement.

For the baseline and the second OCT analysis, the measurements were repeated twice, and the mean of differences for the measurements of the same eye in the same session was 4.5 ± 14.6 *μ*m (range from −19 to 18 *μ*m), the difference was insignificant on a paired *t* test (*P*: 0.7), and the intraclass correlation of these two measurements was statistically significant (*r*: 0.9, *P*: 0.00). A Bland-Altman plot of difference against mean choroidal thickness showed no significant change in intrasession variability for the range of choroidal thickness measurements seen in healthy subjects, and the plot is shown in Figure 1. Although the mean diurnal variation in all groups was −0.03 ± 14.7 and the difference was insignificant, the magnitude of change ranged from −55 *μ*m to 47 *μ*m; therefore, some of the patients showed diurnal variation, and as such six remained unchanged (mean of change was 0.00 *μ*m), forty showed a decrease of mean 11.2 (9.5) *μ*m, and thirty-nine showed an increase of mean 11.5 (11.07) *μ*m.

The eyes were grouped into two subgroups according to the sex for the purpose of additional subanalyses. The CT thickness and the diurnal magnitude were analysed. The mean CT at 9 AM and 4 PM and the magnitude of change of female patients (mean AL: 22.9 mm) were 327.5 (53.8) *μ*m, 320.1 (52.1) *μ*m, and 2.4 (14.5) *μ*m; male patients (mean AL: 23.5 mm) were 293.1 ± 72.2 *μ*m, 295.9 ± 70 *μ*m, and −2.8 ± 14.8 *μ*m, respectively. The mean CT at 9 AM and 4 PM was statistically insignificant between groups when measurements were adjusted according to the AL (*P*: 0.7, *P*: 0.6 resp.), and the magnitude of diurnal change was insignificant in both groups (*P*: 0.3, *P*: 0.2 resp.).

**Figure 2:**
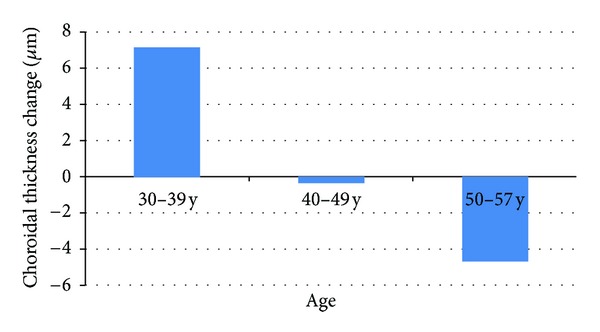
The magnitude of diurnal change according to age group.

There was a weak but statistically significant negative correlation between the magnitude of diurnal change and age (*r*: −0.27, *P*: 0.01). For additional analyses, the eyes were grouped according to age as Group 1 (30–39 years, *n*: 19), Group 2 (40–49 years, *n*: 41), and Group 3 (50–57 years, *n*: 25). The magnitude of change was +7.1 (11.8), −0.3 (15.7), and −4.7 (13.4), respectively and shown in Figure 2. The diurnal variation in Group 1 and Group 3 was significant (*P*: 0.02, *P*: 0.03) while it was insignificant (*P*: 0.7) in Group 2 with a paired *t*-test. There was a statistically significant difference between groups regarding the diurnal variation with Kruskal-Wallis test (*P*: 0.00). The correlation between axial length and diurnal variation was insignificant with Pearson correlation (*r*: −0.19; *P*: 0.3).

## 4. Discussion

In this study we measured the choroidal thickness twice a day, during working hours (early in the morning and late in the afternoon), using EDI spectral domain OCT in the healthy emetropic Turkish population, to investigate diurnal variation. In a study by Ikuno et al. [14], two out of eighty-six eyes were excluded due to poor image quality because of poor fixation by 1060 nm HP-OCT, which is based on swept-source OCT technology. Chen et al. [21] reported that one subject was excluded because of a poor-quality scan due to poor fixation by EDI spectral domain OCT. Manjunath et al. [17] reported that in one of four eyes, the corneoscleral boundary was unclear with Cirrus HD-OCT due to a suboptimal number of averaged OCT B scans, the lack of eye tracking software, and the potential for eye movement during imaging. Our results were comparable with previous studies that, in spite of using high-resolution protocol, an eye tracking system, and ART function, due to inadequate fixation or densely pigmented retinal pigment epithelium, the corneoscleral junction was identified clearly only in 85 percent of eyes for both measurements, and both eyes of the same patient were included in 70 percent of patients in our study. Although swept-source OCT can obtain high-quality images over a greater imaging depth as compared with SD-OCT, corneoscleral junction can be identified with both machines. But due to the poor fixation or densely pigmented retina pigment epithelium, the corneoscleral junction could not be assessed adequately with both of the devices. Ikuno et al. [14] used swept-source OCT and reported exclusion of 2 eyes because of this reason. But in the study of Usui et al. [18], they did not report such a ratio. Besides, the eye tracking system of Heidelberg spectral domain OCT enables us to measure the exact same location over the fovea. Since the choroid does not have an uniform structure for the assessment of diurnal variation, the exact same location had to be remeasured for the accurate results. This is the superiority of SD-OCT over swept-source OCT and the advantage of this study.

The mean choroidal thickness in our study was 308.7 *μ*m, ranging between 140 *μ*m and 496 *μ*m. In a study by Usui et al. [18], the mean CT was 280.3 *μ*m measured by HP-OCT (Topcon), but the population was younger and more myopic. Tan et al. [19] reported a mean CT of 372.2 *μ*m measured by SD-OCT in a younger age group, but the RE was similar. Toyokawa et al. [22] reported a mean CT of 308.5 *μ*m, similar to our study, but the mean age was older (62.6 years). In the study by Ikuno et al. [14], the mean choroidal thickness was 354 *μ*m by 1060 nm HP-OCT, the patients had a wider range of age (23–88 years old), and the mean RE was −1.9 ± 2.3 D. Our results were higher than the 287 ± 76 *μ*m measured with Heidelberg SD-OCT in a study of thirty-four healthy American subjects that was reported Margolis and Spaide [13]. Li and colleagues [15] reported a mean subfoveal choroidal thickness in ninety-three Danish university students measured by Heidelberg SD-OCT at 342 ± 118 *μ*m, in a younger age group (24.9 years old) whose RE was −1.43 D. It has been reported that CT varies according to age and AL. In previous studies by Margolis and Spaide [13] and Ikuno et al., [14], there were decreases of 15 *μ*m and 14 *μ*m in CT, respectively, for every 10 years. Also it has been reported that choroidal thickness changes −58.2 [15] to −22.4 [1] for every increment in 1 mm of AL. Therefore, the differences in mean choroidal thickness between the studies may result from differences in the study population, OCT machine used, and measuring software.

To the best of our knowledge, this is the largest study of diurnal variations in healthy emetropic patients. We did not observe a significant average diurnal variation in choroidal thickness in our study population. Previous investigators demonstrated significant diurnal variations in CT. Brown et al. [23] used the signal processing technique and found a mean CT of 426 *μ*m and a diurnal change of 59.5 ± 24 *μ*m. Chakraborty and colleagues [24] also used the signal processing technique and reported the findings of diurnal variations in thirty healthy eyes. They showed that choroidal thickness increases progressively from 12 PM to 6 PM and the mean amplitude of thickness change was 29 ± 16 *μ*m. Usui et al. [18] reported a significant subfoveal choroidal thickness circadian change by using a high penetration OCT (Topcon). The magnitude of change averaged 33 *μ*m in the thirty-eight eyes of nineteen healthy subjects (mean age was 34.8 years), with the thickest at 3 AM and thinnest at 6 PM. However, unlike our study, most of the subjects were at myopic range with a mean RE of −4.4 ± 2.4; the population was younger and the mean baseline CT was thinner than ours. Tan et al. [19] also reported a significant diurnal variation in twelve healthy volunteers (mean age was 30 years and mean RE was −0.46 ± 1.3 D) measured by Heidelberg SD-OCT and averaging 33.3 *μ*m, with the thickest being at 9 AM and the thinnest at 5 PM, which is comparable to the findings of Usui et al. [18]. However, the study population is younger than ours and the mean baseline CT is thicker. Toyokawa et al. [22] reported a significant diurnal variation of 20 *μ*m, with the thickest being in the evening, although the baseline choroidal thickness is the same and the study population was older than ours. In studies by Usui et al. [18] and Tan et al. [19], the mean choroidal thickness decreased during day while Chakraborty et al. [24] and Toyokawa et al.'s [22] population showed an increase. Although these studies used different measurement techniques that consisted of optical biometers and OCT and there were differences in demographic data between groups, they both showed a significant diurnal variation, averaging from 59.5 *μ*m to 33 *μ*m. Although the mean CT difference decreased during the day, six out of thirty-eight eyes in Usui et al.'s [18] study showed an increment and thirty-two showed a decrement from the baseline. The magnitude of diurnal change ranged from −55 *μ*m to 47 *μ*m in this study. Some patients showed an increment, some stayed stable, and others showed a decrement from the baseline, but the average was statistically insignificant. Demographic differences such as age and AL, the differences in the baseline central subfoveal choroidal thickness, and the diversity of OCT machines used in our study and in previous studies [18, 19, 22] may lead to the differences in the study results. On the other hand, Tan et al. [19] also demonstrated that there is a remarkable congruence in the diurnal pattern of individual eyes on different days. However, this and the previous studies [18, 19, 21] showed a significant correlation between the right and left eyes among CT at 9 AM and 4 PM and the magnitude of diurnal change. Therefore, the exact pattern of diurnal variation may vary from person to person and may not be constant in all individuals, but there is harmony between two eyes. In this series, the central macular thickness measurement did not show any variation between two measurement points. This finding is consistent with previous reports [18, 19]. Also there was no correlation between the central macular and choroidal thicknesses.

In previous reports by Li et al. [15], the subfoveal choroid was thicker in men than women and there was a 62 *μ*m difference between groups. We did not find any differences among CT and CT variation in between the two sexes when we adjust the two groups according to AL. To the best of our knowledge, this is the only study analysing diurnal variations in between two sexes.

Previous studies have reported similar negative correlations between choroidal thicknesses with age [13, 16, 17]. Although the diurnal variation of CT was insignificant in all groups in this study, we found differences between decades. In young patients, choroidal thickness tends to increase during the day while the diurnal variation becomes negatively correlated with the age increments. This finding is also comparable with the study by Tan et al. [19], but the older population in the study by Toyokawa et al. [22] showed a significant increase in CT diurnally.

Previous studies reported diurnal variations in AL [16, 19, 24]. We measured AL once in the morning, and we did not analyse AL change in this study. Therefore, the relationship between CT change and AL was made with baseline AL measurements. We did not find any correlation between AL and CT change, which contradicts a study by Tan et al. [19]. Although mean AL values did not differ much in these two studies, it had a narrower interval in our study, which may cause such a discrepancy between the studies.

The main limitations of this study are that systemic factors such as diastolic and systolic blood pressure were not assessed, diurnal IOP and AL change were not analysed, and only healthy emetropic patients with a limited range of age were included. Also the choroidal thickness was assessed during two time points only in working hours and in only one day so the variation during evening and night and the variation on different days were not assessed in this study.

In conclusion, the pattern of diurnal variation of choroidal thickness may remain stable and may show a decrease or increase from the baseline according to age, but there is great harmony between the two eyes. Therefore, when assessing choroidal thickness in the clinic, a patient's individual diurnal variation pattern should be taken into account.

## Figures and Tables

**Table 1:** Demographics of the subjects in the study.

Parameters	Mean (SD)	Range
Age, y	45.2 (7.4)	30–57
Axial length, mm	23.1 (0.8)	21.3–24.9
Refractive error, D	+0.50 ± 0.25	−1.00 to +0.75
IOP, mmHg	15.3 ± 1.9	8–19

SD: standard deviation.

**Table 2:** Choroidal thickness in all groups, RE and LE and central macular thickness at 9 AM, 4 PM, and the magnitude of diurnal change.

	At 9 AM Mean (SD) (range)	At 4 PM Mean (SD) (range)	Diurnal change Mean (SD) (range)	*P*
All eyes, CT, *µ*m	308.7 (64.5)	308.7 (62)	−0.03 (14.7)	0.9
	(145–496)	(140–485)	(−55 to 47)	
All eyes, CMT, *µ*m	271.1 (18.1)	271.3 (17)	−0.17 (3.02)	0.6
	(243–328)	(244–329)	(−8 to 8)	
RE CT, *µ*m	304.9 (64)	305.9 (59)	−0.97 (16.6)	0.9
	(152–444)	(150–423)	(−55 to 47)	
LE CT, *µ*m	312.5 (65.4)	311.6 (65)	0.92 (12.7)	0.9
	(145–496)	(140–485)	(−36 to 36)	

CT: choroidal thickness, CMT: central macular thickness, RE: right eye, LE: left eye.
